# Anxiety and peripheral artery disease

**DOI:** 10.1042/CS20258419

**Published:** 2026-05-18

**Authors:** Jonathan Golledge, Kim G. Smolderen, Shivshankar Thanigaimani

**Affiliations:** 1 Queensland Research Centre for Peripheral Vascular Disease, College of Medicine and Dentistry, James Cook University, Townsville, QLD 4811, Australia; 2 Department of Vascular and Endovascular Surgery, Townsville University Hospital, Townsville, QLD 4814, Australia; 3 Yale School of Medicine, New Haven, Connecticut, USA.

**Keywords:** Anxiety, anxiolytics, cognitive behavioural therapy, exercise therapy, peripheral artery disease, revascularisation

## Abstract

This review pooled past findings on the prevalence and impact of anxiety among patients with peripheral artery disease (PAD). Past studies have mainly assessed anxiety symptoms using questionnaires, with a pooled analysis of 10 studies suggesting anxiety symptoms to be present in 38% of patients with PAD. An analysis of US hospital admission data suggested that the prevalence of a prior diagnosis of anxiety disorder had doubled between 2011 and 2017 in patients undergoing revascularisation to treat PAD. Risk factors identified more frequently among PAD patients with anxiety symptoms included female sex, financial concerns and lack of social support. Anxiety symptoms have also been associated with worse health-related quality of life, more severe leg pain and poor walking ability. Although the long-term impact of anxiety symptoms on PAD outcomes is largely unexplored, one study reported that perceived chronic stress, which is common in anxious patients, was associated with a two-fold increased risk of death. Anxiety symptoms are associated with activation of the hypothalamus-pituitary-adrenal axis and systemic inflammation, which could potentially contribute to excess adverse event risk among PAD patients. Anxiety is frequently observed around the time of surgery, with one randomised trial reporting reduced anxiety symptoms while listening to classical music during revascularisation. Exercise therapy has also been reported to reduce anxiety symptoms in some trials. In conclusion, anxiety symptoms are frequently observed in PAD patients and associated with poor health status. Future research is needed to clarify the association of anxiety symptoms with long-term outcomes and to identify effective interventions to limit anxiety symptoms among PAD patients.

## Introduction

Occlusion or narrowing of the arteries supplying blood to the legs, usually referred to as peripheral artery disease (PAD), affects about 6% of adults [1]. Important risk factors for PAD include older age, diabetes, smoking, hypertension, dyslipidaemia, and chronic kidney disease [1,2]. People with PAD have reduced health-related quality of life, walking impairment, and increased risk of cardiovascular events, including myocardial infarction, stroke, amputation and death [2]. PAD leads to difficulty in walking and is associated with functional and cognitive decline, as has been widely reported [2]. There is, however, increasing recognition that PAD is also frequently associated with a variety of mental health problems, as highlighted by a recent American Heart Association Statement [3]. Of these mental health problems, depression has received most attention being the focus of several past reviews [3–5]. In contrast, anxiety has received very limited attention despite recent research suggesting it affects most people with PAD [6,7]. Given that anxiety disorders have been reported to be causally associated with greater risk of cardiovascular diseases, such as coronary heart disease, there is a need for more focus on anxiety among people with PAD [8]. This article outlines prior research examining the prevalence and consequences of anxiety symptoms in people with PAD. We also discuss the associated risk factors and biomarkers to provide mechanistic insights.

## Methods

This article was prepared as a narrative critical review of the contemporary literature, rather than a systematic review. No strict inclusion criteria were used. Instead, investigations reporting the prevalence of anxiety symptoms in people with PAD, as well as the association of anxiety symptoms with risk factors, quality of life, and complications of PAD were discussed. Furthermore, we sought studies examining potential mechanisms by which anxiety might promote PAD and favour complications of PAD. The PUBMED database was searched for articles published in English between 1 January 2000 and 31 June 2025. Relevant publications were also identified from reference lists of the selected papers. Search terms used included: ‘PAD’, ‘anxiety’, ‘biomarkers’, ‘genome wide association’, ‘mechanisms’, ‘cardiovascular’, and ‘randomised controlled trial’. We focused on human studies published within the last 5 years, including investigations of the prevalence, mechanisms, complications, and outcome of PAD in relation to anxiety symptoms. For each population, the proportion of PAD patients with anxiety was calculated and variance stabilisation was performed using the Freeman–Tukey double arcsine transformation. A random-effects model was applied to account for clinical and methodological heterogeneity between populations, with between-population variance estimated using the restricted maximum likelihood method. Pooled prevalence estimates are presented with 95% confidence intervals, and heterogeneity was quantified using I^2^ and tau^2^. Prevalence of anxiety in men and women were calculated using random-effects models.

## Anxiety symptoms and disorders

Anxiety disorders are a collection of mental health problems characterised by fear and worry affecting usual daily activities [9]. Anxiety disorders recognised by the Diagnostic and Statistical Manual of Mental Disorders and International Classification of Diseases (ICD) include generalised anxiety disorder (GAD), panic disorder, agoraphobia, social anxiety disorder, specific phobia, separation anxiety and selective mutism [9]. These anxiety disorders require formal diagnosis by a psychiatrist, and this has not been a feature of past studies of PAD patients, which in most cases have focused on assessment of anxiety symptoms, such as nervousness, worrying, being afraid and trouble relaxing, using questionnaires. Patients with an anxiety frequently have another mental health disorder, particularly depression [9]. Due to frequent co-occurrence of anxiety and depression, these have often been combined in reports of patients with PAD, which may have obscured the prevalence and importance of anxiety. Other commonly occurring concurrent mental health disorders include bipolar disorder, substance dependence disorder, obsessive-compulsive disorder, and post-traumatic stress disorder [9]. Currently the information on specific anxiety disorders in people with PAD is limited, with the absence of psychiatric diagnostic interview data, with most past studies focusing on assessing symptoms of anxiety using questionnaires or in the case of one study examining past admissions for treatment of an anxiety disorder (Table 1) [6,7,10–33].

**Table 1:** The definition of anxiety and description of population enrolled for the included studies investigating people with PAD

Population	Assessment method	Definition	Proportion of anxious patients	Study
Newly diagnosed or worsening intermittent claudication recruited to the PORTRAIT registry	GAD-2	≥3	169/1078 (15.7%)	[13,17,21,31]
Revascularisation for PAD	HADS-A	Score not cut-off	NR	[14]
Patients with intermittent claudication taking part in telehealth coaching	GAD-7	≥5	599/1687 (35.5%)	[25,26]
Patients with type 2 diabetes and PAD who have undergo partial foot amputation	HARS	≥1	651/785 (82.9%)	[7]
Patients with PAD managed by one vascular surgery service	HADS-A	≥9	31/127 (24.2%)	[10]
US national sample of patients admitted to hospital for revascularisation to treat CLTI	ICD 9 and 10 codes for anxiety disorders	ICD coding	23,044/384,429 (6.0%)	[15]
Patients with intermittent claudication receiving supervised exercise enrolled in the ELECT registry	HADS-A	≥8	NR	[16]
Participants of the MULAN trial examining the effect of classical music on anxiety during endovascular revascularisation for PAD	Numerical rating scale of anxiety during the procedure	Score not cut off	NR	[22]
Participants with intermittent claudication taking part in the ARMEX trial testing arm ergometry versus treadmill exercise	HADS-A	Score not cut off	NR	[20]
Patients with intermittent claudication or CLTI treated with endovascular therapy or medical therapy alone	HADS-A	Score not cut off	NR	[11]
Patients with CLTI having revascularisation	Beck Anxiety and Depression Inventories	≥8	40/82 (48.8%)	[6]
Participants with intermittent claudication taking part in a trial comparing calf raising exercise with walking exercise	Anxiety assessed as part of the CLAU-S quality of life questionnaire	Score not cut off	NR	[32]
Participants with PAD recruited with a group with cardiovascular disease as part of a cluster trial testing a comprehensive secondary prevention program	Goldberg anxiety–depression scale	≥4	402/1220 (33.0%)†	[12]
Participants of a pooled analysis of 3 trials testing naftidrofuryl for treating intermittent claudication	Anxiety assessed as part of the CLAU-S quality of life questionnaire	Score not cut-off	NR	[29]
Patients with CLTI recruited from two Dutch hospitals	Six and 12-item State-Trait Anxiety Inventory	≥22* ≥12**	177/187 (94.7%)*	[23]
Patients undergoing revascularisation for PAD or AAA repair	How worried are you about surgery?	Five-point Likert scale	NR	[18]
Patients undergoing angioplasty for PAD	Perceived degree of calmness-anxiety and Swedish Mood Adjective Check List	Six-graded Likert scale	12/41 (29.3%)	[19]
European patients with coronary artery disease who have prior diagnosis of PAD	HADS-A	Score not cut off	NR	[24]
Patients with intermittent claudication involved in a home exercise program	The Geriatric Anxiety Inventory-Short Form	Score not cut off	NR	[27]
Patients with PAD or coronary heart disease taking part in a 12-week exercise rehabilitation program	HADS-A	Score not cut off	NR	[30]
Registry of Dutch patients with intermittent claudication	HADS-A	≥8	141/557 (25.3%)	[33]
Dutch patients with PAD	HADS-A	≥8	52/181 (28.8%)	[28]
**Overall**			**24,749/389,233 (6.4%)‡**	
**Excluding study defining anxiety based on past hospital admissions to treat anxiety disorders**			**1898/4804 (39.5%)**	

HADS: hospital anxiety and depression scale; HARS: Hamilton anxiety rating scale; GAD: generalised anxiety disorder scale; CLTI: chronic limb threatening ischemia; ELECT: prospective cohort study of patients with intermittent claudication receiving supervised exercise therapy recruited from 10 vascular surgery departments in the Netherlands; AAA: abdominal aortic aneurysm; NR: not reported; *12-item State-Trait Anxiety Inventory assessed anxiety propensity not current anxiety, which was assessed with **6-item State-Trait Anxiety Inventory but not collected at baseline (pre-operatively); † Only 79 of the 1224 (6.5%) enrolled patients had PAD; ‡ Weighted for relative number of patients with PAD in the study reported by Brotons and colleagues [12]; # synonymous with severe anxiety; PORTRAIT: Patient-Centered Outcomes Related Treatment Practices in Peripheral Arterial Disease: Investigating Trajectories.

## Anxiety and the risk of developing peripheral artery disease

Twin studies suggest that the heritability of anxiety disorders is approximately 40%, suggesting that environmental effects stimulate anxiety in individuals with a genetic predisposition for anxiety [34,35]. Mendelian randomisation studies support a causative role for genetic predisposition to anxiety disorder in increasing cardiovascular disease risk, including that of coronary artery disease, although no studies focused on PAD were identified [36,37]. An analysis of the UK biobank and FinnGen suggested that genetic predisposition to anxiety disorders increased the risk of cardiovascular disease by 1.11-fold (95% confidence intervals, CI, 1.07, 1.15, *P* < 0.001) when assessed using inverse variance weighting but not the Mendelian randomisation-Egger method (odds ratio, OR, 1.03, 95% CI 0.92, 1.14, *P* = 0.652) [37]. Another analysis using data from the UK biobank and FinnGen reported that genetic predisposition to anxiety disorders increased the risk of myocardial infarction by 5.04-fold (95% CI 1.45, 17.52, *P* = 0.011) and heart failure by 3.26-fold (95% CI 1.46, 7.25, *P* = 0.004) when analysed by inverse variance weighting [36]. Given that depression has been shown to double the risk of developing PAD in 7 years (Hazard ratio: 2.09, 95% CI 1.09–4.00, *P* = 0.03), it should be noted that anxiety could similarly contribute to PAD risk through shared pathways [38]. Furthermore, the anxiety–PAD link could be influenced by behavioural mediators in people with anxiety such as smoking, physical inactivity, and unhealthy diet [39]. However, no longitudinal cohort or Mendelian randomisation studies examining the association of anxiety with PAD development were identified. Animal testing would be the most feasible method to investigate a possible anxiety–PAD link prior to undertaking human clinical trials. Although no animal studies have directly investigated the association between anxiety and PAD development, validated behavioural tests exist to model anxiety-like phenotype in rodents. The elevated plus maze, open field test and light-dark box assesses the rodents’ innate aversion to open or brightly lit spaces, suggesting an increased anxiety-like behaviour [40]. Social interaction tests quantify anxiety in rodents by assessing their exploratory behaviours when exposed to unfamiliar environment [40]. Other chronic tests such as chronic unpredictable stress and predator stress induce sustained anxiety-like states via neuroendocrine activation, autonomic imbalance and systemic inflammation, thereby modelling key aspects of persistent anxiety disorders. These features can be adapted to investigate a potential anxiety–PAD link by combining with established PAD models to explore shared pathways (inflammation, autonomic dysregulation) or behavioural mediators such as inactivity.

## Assessment and prevalence of anxiety among people with peripheral artery disease

Anxiety has been assessed in patients with established PAD using a variety of different tools, as illustrated in Table 1 [6,7,10–33]. Assessments have largely been performed using questionnaires, with the Hospital Anxiety and Depression Scale (HADS) being the most commonly used [10,11,14,16,20,24,28,30,33]. Other questionnaires used include the GAD scale 2 or 7 [13,17,21,25,26,31], Hamilton anxiety rating scale (HARS) [7], the Beck Anxiety and Depression Inventories [6], the Goldberg anxiety-depression scale [12], the State-Trait Anxiety Inventory [23], and the Geriatric Anxiety Inventory-Short Form [27]. Numerical ratings, including Likert scales and answers to disease-specific quality of life questionnaires, have also been used [18,19,22,29,32]. Finally, ICD codes for hospital admission to treat anxiety disorders have been used [15]. An International Consortium for Health Outcomes Measurement (ICHOM) consensus group recommended using GAD-7 to assess anxiety due to its excellent scope to measure between-individual differences, its availability in multiple languages and its acceptability in the field [41]. It was accepted that GAD-7 was designed to assess generalised anxiety disorders and maybe inappropriate for some specific anxiety disorders, such as agoraphobia. The ICHOM group recommended functioning should also be assessed due to the large amount of evidence indicating that anxiety (and associated depression) impair ability to function. The World Health Organisation Disability Schedule 2.0 was recommended for measuring function [41]. As illustrated in Table 1, GAD-7 has rarely been used to assess anxiety in people with PAD [6,7,10–27,29–33].

**Figure 1:**
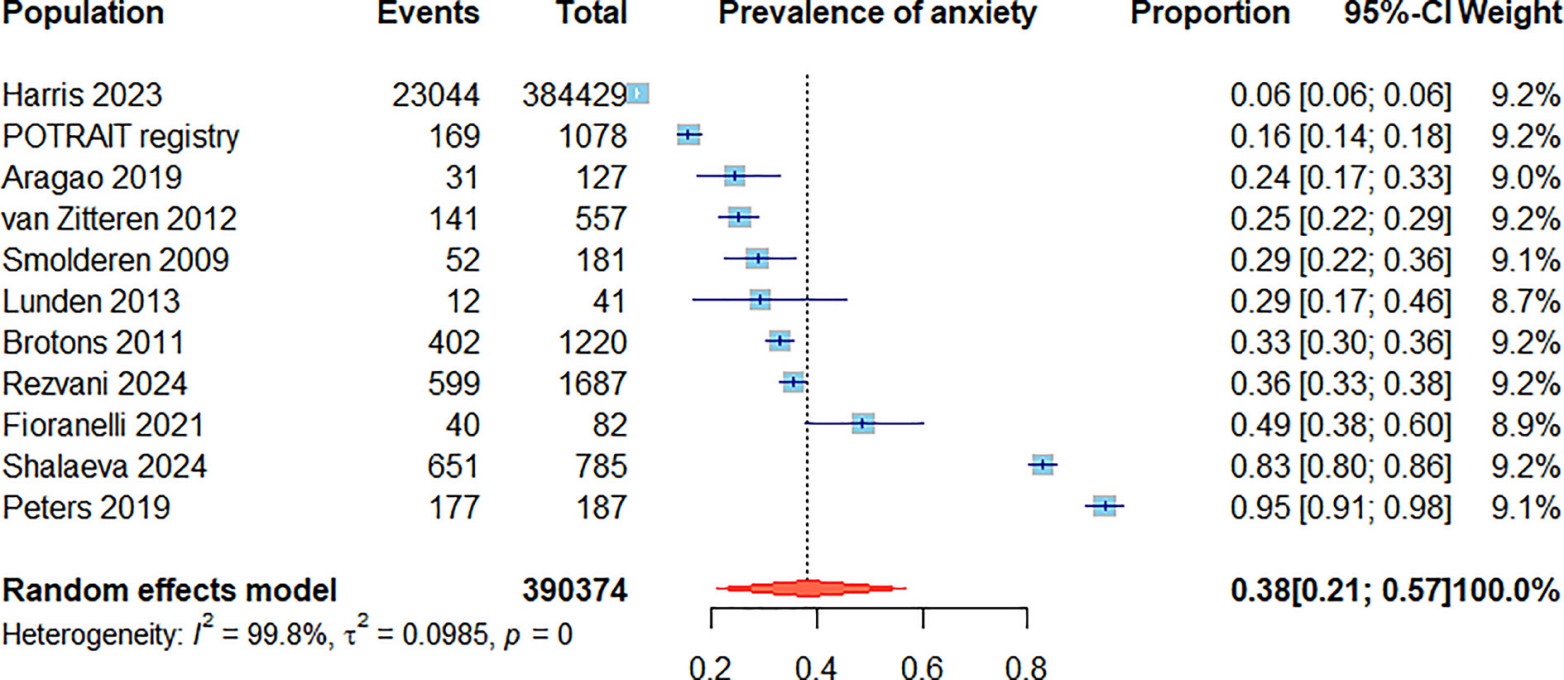
Forrest plot illustrating the mean and 95% confidence intervals of prevalence of anxiety symptoms or anxiety disorder within included studies. Forrest plot illustrating the mean and 95% confidence intervals of prevelance of anxiety symptoms or anxiety disorder within included studies. The diamond represents a pooled summary estimate of the prevalence of anxiety in participants with PAD.

The reported prevalence of anxiety symptoms among patients with PAD has varied from 6.0% to 94.7% depending on population studied, the method of defining anxiety and the timing of the assessment (Table 1) [6,7,10–33]. Considering all the studies identified that reported administering questionnaires to patients with PAD, the mean prevalence of anxiety symptoms was approximately 38.3% (95% CI: 21.2, 56.9) (Table 1 and Figure 1) [6,7,10–27,29–33]. A study of 1687 German patients with intermittent claudication assessed the severity of anxiety symptoms using the ICHOM group recommended GAD-7 scale [26]. Overall, 435 (25.8%) had mild anxiety, 119 (7.1%) moderate anxiety, and 45 (2.7%) severe anxiety. Patients with anxiety had greater walking impairment as assessed by the walking impairment questionnaire than those with no anxiety [26]. In contrast, a study of 785 with patients with type 2 diabetes and PAD who had undergone partial foot amputation reported that 429 (54.6%), 112 (14.3%), 40 (5.1%), and 70 (8.9%) had mild, mild to moderate, moderate to severe, and severe anxiety, respectively, as assessed by the HARS [7]. Of note, severe anxiety was associated with an increased risk of mortality (HR 2.26, 95% CI 1.26, 4.06, *P* = 0.006) after adjusted for other risk factors [7].

The Patient-Centered Outcomes Related Treatment Practices in Peripheral Arterial Disease: Investigating Trajectories (PORTRAIT) registry recruited patients with new onset or worsening intermittent claudication and in the most recent report it was indicated that 169 of the 1078 (15.7%) patients screened positive for clinically relevant anxiety symptoms as assessed by GAD-2 scores ≥3 [13,17,21,31]. A study of Brazilian patients with PAD recruited from a tertiary hospital vascular surgery service found that 31 (24.4%) had anxiety as assessed by the HADS [10]. Anxiety was more common among patients who were smokers and on monthly family income. Harris and colleagues used the US National Inpatient Sample to study history of admission with anxiety disorders among patients with chronic limb threatening ischemia (CLTI) undergoing revascularisation [15]. Overall, 23,044 of the 384,429 patients (6.0%) were identified to have been admitted to hospital for treatment of an anxiety disorder based on ICD coding. The prevalence of past hospital admission with an anxiety disorder increased from 2.9% in 2011 to 7.7% in 2017 for patients undergoing endovascular revascularisation and from 3.3% in 2011 to 7.8% in 2017 for those having surgical revascularisation [15]. A prior anxiety disorder was associated with a longer length of hospital stay and higher cost for the in-patient revascularisation treatment [15].

A study of 237 patients with intermittent claudication treated with supervised exercise therapy found that anxiety symptoms were associated with poorer health-related quality of life as assessed by the Vascular Quality of Life Questionnaire-6 [16]. In a study of 187 patients with CLTI, most (95%) were assessed as prone to anxiety using the 12-item State-Trait Anxiety Inventory [23]. Fioranelli et al. assessed anxiety symptoms in 82 patients with PAD undergoing revascularisation using the Beck Anxiety and Depression Inventories [6]. Preoperatively 25 (30.5%), 11 (13.4%), and 4 (4.9%) had mild, moderate, and severe anxiety, respectively [6]. Thirty days after revascularisation, only 8 of the 50 (16%) patients assessed had anxiety symptoms (7 mild and one moderate). This highlights that anxiety prior to revascularisation is very common.

Brotons and colleagues reported a randomised clinical trial testing the effect of a comprehensive secondary prevention program on the combined outcome of hospital admission to treat cardiovascular disease and death [12]. Overall, 79 of patients randomised had PAD, with the other patients enrolled having coronary heart or cerebrovascular disease. Among the 1220 patients in whom data was reported, 402 (33.0%) had anxiety symptoms at the time of enrolment according to the Goldberg anxiety-depression scale [12].

There have been few studies directly comparing the prevalence and severity of anxiety symptoms in patients with PAD and healthy controls. A study of 159 patients undergoing revascularisation for PAD and 160 sex-matched healthy controls assessed affective temperaments using the temperament evaluation of Memphis, Pisa, Paris, and San Diego-Auto questionnaire (TEMPS-A) and anxiety symptoms using the HADS [14]. Anxious temperament characteristics as measured by TEMPS-A were significantly greater in PAD cases than controls. While it was stated in the abstract that the prevalence of anxiety symptoms was greater in participants with PAD than controls, details of this were not reported in the results [14]. Among patients with PAD, the severity of anxiety symptoms as measured by HADS was negatively correlated with ankle brachial index and positively correlated with the severity of leg pain assessed on a numerical rating scale [14]. A multi-centre study of 8243 European patients with coronary heart disease identified 526 (6.4%) with previously diagnosed PAD [24]. Anxiety as assessed by the HADS was significantly greater in patients with diagnosed PAD (mean 5.86, SD 4.14) as compared to those without this diagnosis (5.36, 3.78, *P* < 0.05) [24].

Overall, these findings illustrate that symptoms of anxiety affect most patients with PAD, with prevalence varying depending on the population, assessment method and disease severity, and being consistently associated with poor outcomes including walking impairment, reduced quality of life and elevated mortality risk. These findings highlight the need for routine screening of risk factors and health status for anxiety in PAD patients using integrated psychological support and targeted interventions.

## Association of anxiety with risk factors and health status

A few studies have compared risk factors between PAD patients who did and did not exhibit symptoms of anxiety (see Table 2) [21,26]. Odu and colleagues assessed 1078 patients with new or worsening intermittent claudication enrolled in the PORTRAIT registry with the GAD-2 questionnaire [21]. Patients assessed as anxious (GAD-2 score ≥3) were significantly younger, more likely to be female, current smokers and be receiving medication for depression, than those found to not be anxious (GAD-2 score <3; Table 2) [21]. Surprisingly, coronary heart disease (defined as previous coronary artery revascularisation) was significantly more common in non-anxious than anxious patients. At entry and 3-, 6- and 12-months follow-up, health status as assessed by the Peripheral Artery Questionnaire (PAQ) was significantly poorer in anxious compared to non-anxious patients. Revascularisation was reported to lead to significantly greater improvement in PAQ scores in anxious as compared to non-anxious patients [21].

**Table 2:** Studies comparing risk factors among people with peripheral artery disease with and without anxiety

Study	Odu et al. [21]		Rezvani et al [26]		Harris et al [15]	
	Anxious (*n* = 169)	Not anxious (*n* = 909)	Anxious	Not anxious	Anxious (*n* = 23,044)	Not anxious (*n* = 361,385)
Age	63.6 (9.2)*	67.8 (9.3)*	63.9 (9.1)*	67.6 (8.1)*	66.3 (12.6)*	69.2 (12.1)*
Female sex	91 (53.8)*	317 (34.9)*	226/590 (38.3)*	296/1073 (27.6)*	12,144 (52.7)*	136,242 (37.7)*
Caucasian	136 (80.5)	759 (83.5)	575/575 (100)	1,051/1,051 (100)	16,615 (72.1)*	220,806 (61.1)*
African–American	22 (13.0)	121 (13.3)	0	0	2,512 (10.9)*	43,366 (18.2)*
Other	11 (6.5)	29 (3.2)	0	0	2,512 (10.9)	49,871 (13.8)
Higher education	115 (68.0)	654 (71.9)	NR	NR	NR	NR
Health insurance	167 (98.8)	902 (99.2)	NR	NR	22,537 (97.8)	352,712 (97.6)
Current smoking	89 (52.7)*	307 (33.8)*	NR	NR	12,375 (53.7)*	153,950 (42.6)*
Heart failure	17 (10.1)	86 (9.5)	107/533 (20.1)*	151/999 (15.1)*	1,175 (5.1)	24,213 (6.7)
CHD	53 (31.4)*	335 (36.9)*	94/541 (17.4)*	123/1012 (12.2)*	11,983 (52.4)	186,113 (51.5)
Hypertension	138 (81.7)	727 (80.0)	419/566 (74.0)	800/1048 (76.3)	19,449 (84.4)	298,142 (82.5)
Diabetes	60 (35.5)	294 (32.3)	136/543 (25.0)	301/1023 (29.4)	8,780 (38.1)	129,014 (35.7)
CVD	19 (11.2)	96 (10.6)	64/541 (11.8)*	84/1014 (8.3)*	NR	NR
Dyslipidemia	137 (81.1)	722 (79.4)	334/549 (60.8)	628/1037 (60.6)	13,665 (59.3)	189,366 (52.4)
CKD	17 (10.1)	100 (11.0)	NR	NR	5,646 (24.5)	110,584 (30.6)
CLD	41 (24.3)	147 (16.2)	119/547 (21.8)*	150/1023 (14.7)*	7,927 (34.4)*	80,227 (22.2)*
Depression or depressive symptoms	59 (35.5)*	133 (14.6)*	523/594 (88.0)*	286/1089 (26.3)*	NR	NR
Stress symptoms†	109 (64.5)*	228 (25.4)*	NR	NR	NR	NR

Data reported for Rezvani and colleagues is based on analysis of the open access data file provided as part of their publication [26]. *P < 0.05; CHD: coronary heart disease; CVD: cerebrovascular disease including stroke and/or transient ischemic attack; CKD: chronic kidney disease; CLD: chronic lung disease; definitions of anxiety used: Generalised anxiety disorder-2 scale (GAD-2) ≥3 [21], GAD-7 ≥5 [26], ICD-9, or 10 codes for hospital admission with an anxiety disorder (generalised anxiety disorder, panic disorder or other anxiety disorder) [15], † assessed by the perceived stress scale-4 (PSS-4) ≥6 [21].

Rezvani and colleagues examined 1687 patients with intermittent claudication taking part in a randomised controlled trial testing a telephone exercise coaching program including remote monitoring of physical activity [26]. The authors provided open access data which was analysed to compare risk factors in patients determined to be anxious based on GAD-7 scores ≥5 compared to those with lower scores (Table 2). Anxious patients were significantly younger, more likely to be female, have a history of heart failure, coronary heart disease, cerebrovascular disease, chronic lung disease and symptoms of depression (Table 2). Walking impairment defined with the walking impairment questionnaire was positively correlated with the severity of anxiety symptoms based on GAD-7 scores [26]. Among patients with CLTI undergoing revascularisation included in the US National Inpatient Sample, prior admission with an anxiety disorder was significantly more common in younger people, women, caucasians, current smokers and those with a history of chronic lung disease (Table 2) [15].

In the PORTRAIT registry, patients with anxiety were significantly more likely to have perceived stress, with 65% of anxious patients having perceived stress according to a Perceived Stress Scale-4 (PSS-4) score ≥6 (Table 2) [21]. Both anxiety and stress were associated with low social supported as assessed by the Enhancing Recovery in Coronary Heart Disease Patients (ENRICHD) Social Support Inventory (ESSI) [13]. Patients with low social support had a lower disease-specific and generic health status at baseline and 12 months. In an analysis of 957 patients with new or worsening intermittent claudication recruited to the PORTRAIT registry, 28.7% reported high levels of perceived stress as assessed by the perceived stress scale-4 score ≥6 [31]. Among US patients recruited to the PORTRAIT registry, 21% reported having no or incomplete health insurance [17]. These patients had more severe symptoms of anxiety, stress, and depression than those with complete health insurance [17].

**Figure 2:**
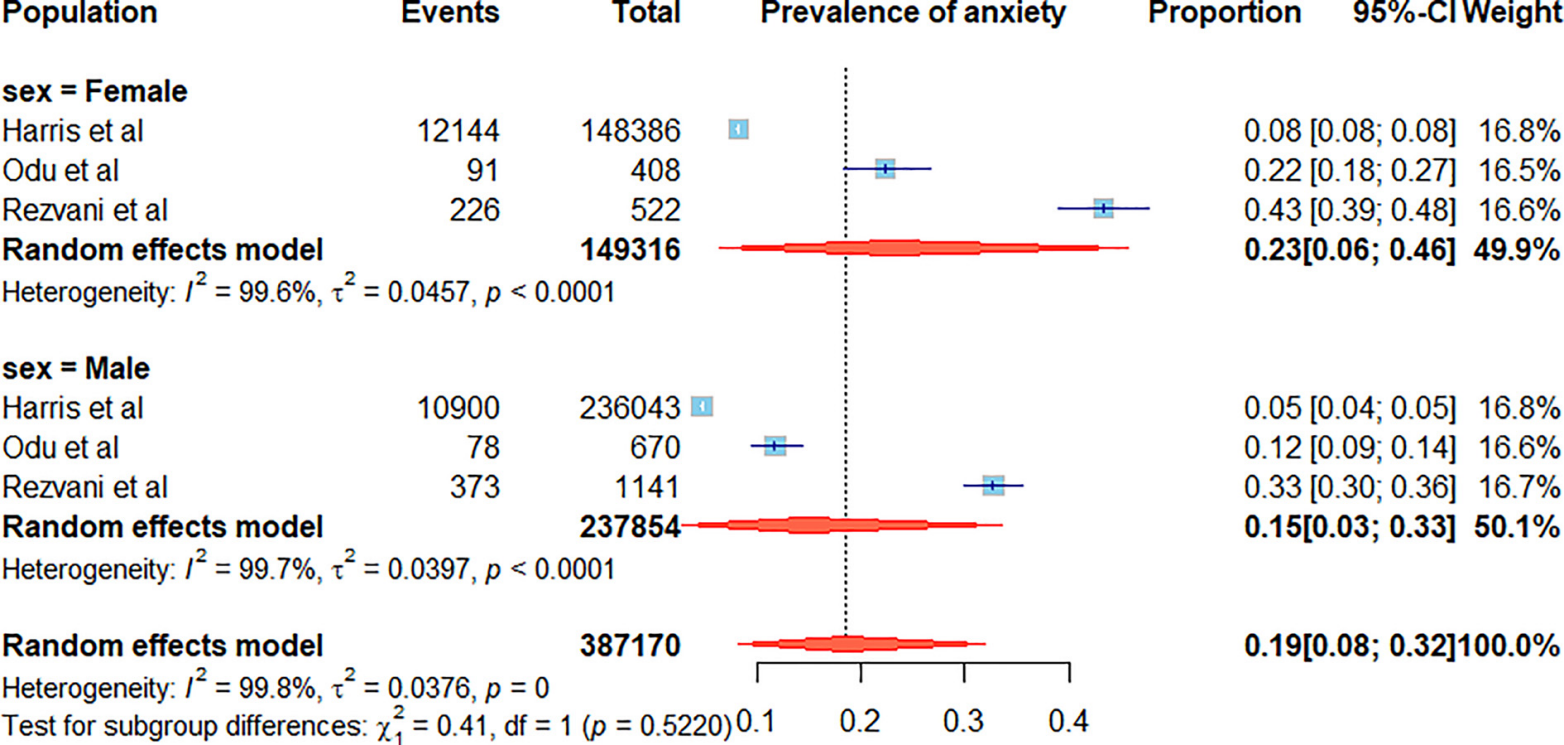
Forrest plot illustrating the mean and 95% confidence intervals of prevalence of anxiety symptoms or anxiety disorder in men and women within included studies. Forrest plot illustrating the mean and 95% confidence intervals of prevalence of anxiety symptoms or anxiety disorder in men and women within included studies. The diamond represents a pooled summary estimate of the prevalence of anxiety.

Van Zitteren and colleagues defined anxiety using the HADS in Dutch patients with intermittent claudication [33]. Patients with occlusive disease involving the femoral, popliteal, and tibial arteries were less likely to have anxiety (69, 19.4%) as compared to patients with aorto-iliac occlusive disease (72, 35.6%, *P* < 0.01) [33]. Smolderen and colleagues studied anxiety in 181 Dutch patients with PAD using HADS [28]. Patients with pain at rest had significantly greater anxiety than asymptomatic patients or those with leg pain [28]. Similarly, Chyrek-Tomaszaewska and colleagues found a significant correlation between more severe anxiety as assessed by HADS scores and more severe pain at presentation, as measured by a numerical rating scale in 157 patients admitted to hospital for revascularisation to treat PAD [14]. Meta-analysis of studies reporting sex-stratified data suggested that females (mean prevalence: 22.9%, 95% CI: 6.4, 45.7) had a higher proportion of anxiety prevalence compared to males (mean prevalence: 14.5%, 95% CI: 2.7, 33.5), although not statistically different (*P* = 0.522) (Figure 2).

Overall, these findings suggest that anxiety is more commonly present in younger female patients with financial concerns and lack of social support. Anxiety is associated with worse health-related quality of life, more severe pain, and poorer walking ability.

## Association of anxiety with complications of peripheral artery disease

Anxiety has been associated with both increased and reduced cardiovascular mortality in the general population and in people with coronary heart disease [42–44]. For example, among Vietnam veterans anxiety was associated with increased cardiovascular mortality [44], while in the Latvian general population [42], and German patients having percutaneous coronary interventions [43], anxiety was associated with reduced cardiovascular mortality. A 2020 meta-analysis of 17 studies found anxiety was independently associated with an increased risk of mortality (adjusted risk ratio 1.21, 95% CI 1.07, 1.37, *P* = 0.002) and increased risk of major adverse cardiovascular events (adjusted risk ratio 1.47, 95% CI 1.24, 1.74, *P* < 0.001) in patients with acute coronary syndrome [45]. In contrast, relatively few studies have examined the association of anxiety with the complications of PAD (Table 3) [7,15]. Shalaeva and colleagues studied 785 patients with PAD and diabetes after partial foot amputation [7]. Overall, 133 (16.9%) had died after one year of follow-up. Thirty of the 70 (42.9%) patients with severe anxiety had died within one year, as compared to 26 of the 134 (19.4%) patients with no anxiety. After adjusting for other risk factors, severe anxiety at baseline was associated with a significantly higher risk of death (hazard ratio 2.26, 95% CI 1.26, 4.6, *P* = 0.006) [7]. Harris and colleagues reported that prior admission with an anxiety disorder was associated with significantly longer length of hospital admission and higher cost following endovascular revascularisation for CLTI [15]. Furthermore, among patients younger than 65 years prior admission with anxiety disorder was associated with a higher risk of major amputation (see Table 3) [15]. Chronic stress is very commonly associated with anxiety and in one report from the PORTRAIT registry, chronic stress was associated with a two-fold increased risk of mortality after adjusting for other risk factors (Table 3) [46]. Peters and colleagues followed 187 Dutch patients with CLTI of whom 177 (94.7%) had anxiety symptoms as assessed by the State-Trait Anxiety Inventory. There was no significant difference in amputation-free survival over 12 months between patients with and without anxiety but there was approximately 50% loss to follow-up and the analysis was not sufficiently powered to rule out a moderate effect [23].

**Table 3:** Association of anxiety with complications of peripheral artery disease

Population	Number	Dependent variable	Outcome	Unadjusted association	Adjusted association
Patients with PAD and diabetes after partial foot amputation	785	Severe anxiety	Mortality	NR	HR 2.26 (95% CI 1.26, 4.06; *P* = 0.006)* [7]
Patients with newly diagnosed or worsening intermittent claudication	765	Chronic stress†	Mortality		HR 2.12 (95% CI 1.14, 3.94; *P* = 0.02)‡ [46]
Patients with CLTI admitted for endovascular revascularisation	245,507	Prior admission for treatment of anxiety disorder	Median length of hospital stay Median cost Major amputation In-hospital mortality		9 versus 8 days, *P* < 0.001# $109,496 vs $102,324, *P* < 0.001# OR 1.13 (95% CI 0.98, 1.13)# OR 0.78 (95% CI 0.54, 1.10)# [15]
Patients <65 years with CLTI admitted for endovascular revascularisation	NR	Prior admission for treatment of anxiety disorder	Major amputation		OR 1.34 (95% CI 1.10, 1.64)# [15]

CI: confidence interval; NR: not reported; *reported for severe anxiety defined by Hamilton anxiety rating scale >30 and adjusted for age, sex, HbA1c, diabetes duration, foot infection complications, ankle brachial index, obesity, previous myocardial infarction, coronary revascularisation, smoking, revised cardiac risk index, non-adherence to prescribed medications, non-compliance to lifestyle recommendations, depression symptoms.† defined as Perceived Stress Scale-4 (PSS-4) ≥6 at 2 or more follow-up assessments; ‡ adjusted for age, sex, race, heart failure, prior myocardial infarction, smoking, diabetes, hypertension, low income, education, anti-platelet and statin prescription, revascularisation and ankle brachial index; # Adjusted for age, sex, race, insurance, income, comorbidities and illness severity.

Overall, these preliminary findings suggest anxiety is associated with worse outcome in people with PAD and might represent a treatment target to reduce PAD complications.

## Mechanisms by which anxiety might worsening outcome in people with peripheral artery disease

**Figure 3:**
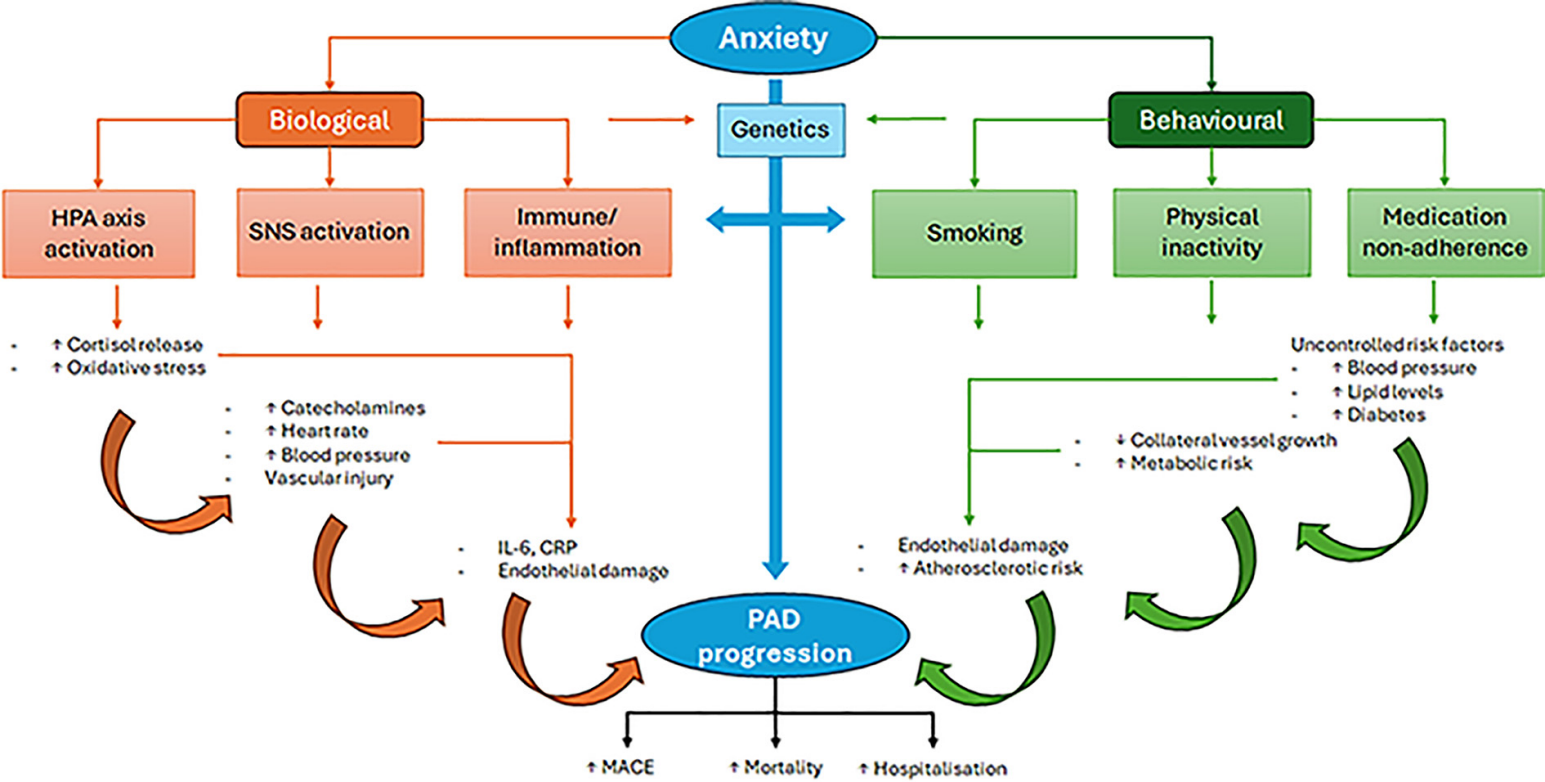
Mechanistic pathways linking anxiety to PAD progression and outcomes. Mechanistic pathways linking anxiety to PAD progression and outcomes. HPA axis: hypothalamic-pituitary-adrenal axis, SNS: sympathetic nervous system, IL-6: interleukin-6, CRP: C-reactive protein, PAD: peripheral artery disease

Currently the impact of anxiety on patients with PAD is poorly understood but there is evidence from other populations that anxiety may influence the development and outcome of PAD by a range of mechanisms, as outlined below (Figure 3).

### Genetics

Mendelian randomisation is believed to provide data comparable to a randomised controlled clinical trial and therefore provide causality inferences. Given the established role of genetic predisposition in the development of anxiety disorder, there is increasing focus on using genome-wide association, Mendelian randomisation and pharmacogenomic methods to try and understand how anxiety contributes to disease and to identify new treatment targets [47–50]. Genome-wide association studies have identified more than 50 anxiety-associated single nucleotide polymorphisms and combined these in polygenic risk scores [51]. A recent analysis of data from 1.2 million people participating in multiple international biobanks identified 51 anxiety associated genetic loci [51]. Combining this with transcriptome and proteome-wide data the authors proposed several mechanisms by which the identified genetic loci could influence anxiety development through high fructose production and changes in brain structure [51]. Using these genetic proxies of anxiety in Mendelian randomisation analyses, recent investigations have reported causal roles for anxiety in increasing risk of coronary heart disease, myocardial infarction, heart failure, hypertension, obesity and heart rate variability (Table 4) [36,52,53]. Mendelian randomisation analyses have also associated multiple gut microbiota and sedentary behaviour with increased risk of anxiety (Table 4) [54–56]. These findings illustrate that a combination of shared risk factors, such as obesity, hypertension, physical activity and microbiome changes likely contributed to the increased risk of cardiovascular disease in people with anxiety disorders.

**Table 4:** Findings from Mendelian randomisation studies investigating mechanisms by which anxiety might influence peripheral artery disease development and complications

Study design	Data source	Findings	Ref.
Two-sample MR analysis using Inverse-variance weighted and medication analyses	European ancestry cohorts	Causal effect of anxiety on hypertension (OR 1.03, 95% CI 1.01, 1.04, *P* < 0.001) and obesity (OR 1.52, 95% CI 1.04, 2.21). 17.4% (95% CI 5.3%–29.5%) of anxiety’s effect on hypertension risk mediated by adiposity	[52]
Two-sample bidirectional MR	FinnGen, Pan-UKBB, and PGC	Gut microbiota implicated as causal in anxiety, *Actinobacteria [p]* (OR 0.88, 95% CI 0.82, 0.95, *P* = 0.002), *Bifidobacteriales* [*o*] (OR 0.86, 95% CI 0.77, 0.96, *P* = 0.009), *Bifidobacteriaceae* [*f*] (OR 0.86, 95% CI 0.77, 0.96, *P* = 0.009), *Eubacteriumrectalegroup* [*g*] (OR 0.90, 95% CI 0.81, 0.99, *P* = 0.026), *ClostridialesvadinBB60group* [*f*] (OR 0.92, 95% CI 0.86 to 0.98, *P* = 0.013), and *Parasutterel* [*g*] showed a protective effect against anxiety. *Allisonella* [*g*] (OR 1.06, 95% CI 1.02 to 1.10, *P* = 0.005), and *Alistipes* [*g*] (OR 1.13, 95% CI 1.05 to 1.22, *P* = 0.002) were positively correlated with anxiety.	[55]
One-sample MR	All of US Research Program	Anxiety polygenic risk score independently associated with a measure of pulse rate variability	[53]
Bidirectional MR	MiBioGen consortium	Anxiety implicated gut microbiota *Eubacteriumbrachygroup* (OR = 1.01, 95% CI, 1.00,1.01, *P* = 0.0225), *Coprococcus3* (OR = 1.02, 95% CI, 1.01, 1.03, *P* = 0.0065), *Enterorhabdus* (OR = 1.01, 95% CI, 1.00, 1.02, *P* = 0.0108), *Oxalobacter* (OR = 1.01, 95% CI, 1.00, 1.01, *P* = 0.0231), and *Ruminiclostridium6* (OR = 1.01, 95% CI, 1.01, 1.02, *P* = 0.0019); *Blautia* (OR = 0.98, 95% CI, 0.97, 0.99, *P* = 0.0056), *Butyricicoccus* (OR = 0.99, 95% CI, 0.97, 0.99, *P* = 0.0233), *ErysipelotrichaceaeUCG003* (OR = 0.99, 95% CI, 0.98, 0.99, *P* = 0.0381), and *Parasutterella* (OR = 0.99, 95% CI, 0.98, 0.99, *P* = 0.0478).	[56]
Two-sample MR	UK biobank and FinnGen	Causal association between anxiety disorders and increased risk of CHD (OR 4.50, 95% CI 1.78, 11.38, *P* = 0.002), MI (OR 5.04, 95% CI 1.45, 17.52, *P* = 0.011), and HF (OR 3.26, 95% CI 1.46, 7.25, *P* = 0.004)	[36]
Two-sample bidirectional MR	UK biobank	Genetically instrumented higher sedentary time associated with higher odds of anxiety (OR 2.59, 95% CI 1.10, 4.60)	[54]

MR: mendelian randomisation; PGC: Psychiatric Genomics Consortium; Pan-UKBB: The Pan-ancestry genetic analysis of the UK Biobank; CHD: coronary heart disease; MI: myocardial infarction; HF: heart failure; OR: Odds ratio; CI: Confidence interval.

### Neurophysiological mechanisms

Anxiety has long been proposed to lead to activation of the hypothalamic-pituitary-adrenal (HPA) axis leading to increased circulating cortisol and angiotensin II and raised heart rate [57]. A study of 145 healthy women aged between 50 and 64 years found that higher levels of hair cortisol and cortisone were associated with higher risk of cardiovascular events as estimated by the SCORE2 algorithm [58]. Hair cortisol levels were correlated with Perceived Stress Scale score (*r* = 0.170, *P* = 0.041), Pittsburgh Sleep Quality Index score (*r* = 0.181, *P* = 0.030) and level of psychological stress as measured by the perceived stress scale (*r* = 0.170, *P* = 0.041). Hair cortisol levels were significantly correlated with systolic (*r* = 0.246, *P* = 0.003) and diastolic (*r* = 0.227, *P* = 0.006) blood pressure. Hair cortisone levels were significantly correlated with body mass index (*r* = 0.307, *P* < 0.001), waist circumference (*r* = 0.344, *P* < 0.001), systolic (*r* = 0.271, *P* < 0.001), and diastolic (*r* = 0.276, *P* < 0.001) blood pressure [58]. Anxiety has also been associated with detrimental changes in vasomotor tone. One study of women with coronary artery spasm but no obstructive lesions suggested that during a mental stress test involving anger recall anxiety, frustration and feeling challenged inversely correlated with vasoconstriction as measured by a peripheral arterial tonometric plethysmography device [59]. Activation of the sympathetic nervous system in patients with anxiety have been associated with changes in gastro-intestinal function including changes in intestinal flora [55–57]. Skin conductance measures electrical conductivity of skin on the fingers and is believed to be a sensitive measure of sympathetic nervous system activity. Anxiety and post-traumatic stress disorder symptoms are common following medical diagnoses. Meinhausen and colleagues studied 64 patients after being diagnosed with a stroke or transient ischemic attack [60]. There was a significant correlation between in-hospital skin conductance to recalling the trauma of stroke or transient ischemic attack and higher-order fear-related symptoms (*r* = 0.30, *P* = 0.016), as well as lower-order fear-related symptoms of anxious arousal (*r* = 0.27, *P* = 0.035) and avoidance (*r* = 0.25, *P* = 0.043), as assessed one month later [60]. Overall, these findings suggest anxiety can lead to a recurrent state of activation of stress-related neurophysiological pathways.

### Biomarkers

In some populations anxiety has been associated with raised circulating levels of a number of biomarkers implicated in cardiovascular disease, including high C-reactive protein, neutrophil count, lymphocyte count, neutrophil lymphocyte ratio, tissue inhibitor of metalloproteinase-4, intercellular adhesion molecule-1 and neutrophil gelatinase-associated lipocalin and low haemoglobin (Table 5) [61–66]. A U-shaped association of anxiety with myeloperoxidase was reported in a large Chinese population [67]. However, not all studies have found associations of circulating inflammatory biomarkers with anxiety [61,68]. Buto and colleagues examined the association of symptoms of anxiety assessed with the State-Trait Anxiety Inventory and high sensitivity C-reactive protein and interleukin-6 in 244 patients aged 25 to 60 years old who had suffered a myocardial infarction between 1-week and 8-months previously [68]. There was no significant association between high sensitivity C-reactive protein and interleukin-6 levels with severity of anxiety symptoms. In contrast, levels of high sensitivity C-reactive protein and interleukin-6 were correlated with post-traumatic stress disorder re-experiencing symptom cluster assessed by the civilian version of the post-traumatic stress disorder checklist [68]. A study of 52 patients with acute coronary syndrome used liquid chromatography–mass spectrometry to identify metabolites differentially expressed in participants with and without anxiety as assessed by the HAS [69]. Thirty-nine metabolites related to tryptophan metabolism, glycerophospholipid metabolism, pentose phosphate metabolism, pyrimidine metabolism, and pentose and glucuronate interconversion were differentially expressed in participants that did and did not have anxiety [69]. Gou and colleagues studied 333,017 adults without a prior diagnosis of mental health disorders [70]. Using 10 biomarkers selected to reflect metabolic, cardiovascular and inflammatory dysregulation, they reported that those with a higher biomarker score had an increased risk of developing an anxiety disorder (Table 5) [70]. In contrast to these findings, circulating high levels of Lipoprotein(a), an established cardiovascular risk factor, and galectin-3, a protein involved in cell adhesion and cell-matrix interactions, have been associated with reduced severity of anxiety in some populations (Table 5) [71,72]. A study of US marines reported that body mass reduction in response to a survival training program was correlated with anxiety severity [73]. A meta-regression analysis of eight studies including 1179 participants suggested that brain-derived neurotropic factor levels were lower in those with anxiety disorders compared to those without (standardised mean difference, SMD −0.94, 95% CI −1.75, −0.12). However, the results varied depending on whether measured from plasma (SMD −1.31, 95% CI −1.69, −0.92) or serum (SMD −1.06, 95% CI −2.27, 0.16), or type of mental health disorder including post-traumatic stress disorder (SMD −0.05, 95% CI −1.66, 1.75) or obsessive-compulsive disorder (SMD −2.33, 95% CI −4.21, −0.45) [74]. Overall, these studies suggest inconsistent associations of anxiety with biomarkers.

**Table 5:** Biomarkers and pathways by which anxiety might promote cardiovascular events

Biomarker/ pathway	Population	Marker assessment	Anxiety assessment	Association with anxiety	Reference
Myeloperoxidase	30,418 Chinese adults	Plasma	Self-rating anxiety scale ≥50 defined as clinically relevant anxiety symptoms	U-shaped association	[67]
Lipoprotein(a)	670 cases and 1340 controls from Nanchang, China	Serum	HARS*	Low lipoprotein (a) associated with greater risk of anxiety (OR 1.23, 95% CI 1.04, 1.46, Q1 compared to Q4)	[71]
High sensitivity C-reactive protein	9704 adults (35–65 years) from Mashhad stroke and heart atherosclerotic disorder cohort study	Serum	Beck’s Anxiety Inventory*	Increased anxiety score associated with high CRP	[64]
Tissue inhibitor of metalloproteinase-4	Nested case-control study of 775 adults (24 years old)	Plasma	Clinical interview schedule revised to defined GAD	High TIMP-4 associated with anxiety (OR 1.43, 95% CI 1.19, 1.72)	[63]
Neutrophil gelatinase-associated lipocalin	150 patients with acute ischemic stroke	Serum	HADS ≥8	Higher NGAL levels associated with anxiety (OR 1.28, 95% CI 1.09, 1.51)	[65]
Allostatic load biomarkers glucose, total cholesterol, HDL, HbA1C, insulin-like growth factor 1, CRP (Plus WHR, BMI, SBP, DBP)	333,017 adults from UK biobank	Combination of blood and anthropometric biomarkers	Diagnoses of anxiety disorder using ICD-10 codes	Higher allosteric load associated with higher risk of anxiety (HR 1.30, 95% CI 1.23, 1.38)	[70]
3-hydroxyanthranilic acid	Colorectal cancer survivors	Plasma	Anxiety severity assess with the HADS	Higher 3-hydroxyanthranilic acid associated with lower HADS anxiety scores	[66]
Intercellular adhesion molecule-1	Patients with breast cancer on aromatase inhibitors	Not reported	Anxiety severity assessed with GAD-7	Significant positive correlation between ICAM-1 levels and GAD-7 scores	[61]
Galectin-3	Adults 50–85 years with risk factors for cardiovascular disease or heart failure	Serum	Anxiety severity assess with the HADS	Significant negative correlation between galectin-3 concentration and HADS scores (*r* = −0.078, *P* = 0.005). Association independent of sex, age, BMI, eGFR, walking distance, SF-36 and diastolic dysfunction grade	[72]
Metabolome	Patients with ACS with or without anxiety that were matched for risk factors	Plasma	HARS score ≥14	39 metabolites differentially expressed, e.g. serotonin highlighting difference in tryptophan metabolism, glycerophospholipid metabolism, pentose phosphate metabolism, pyrimidine metabolism, and pentose and glucuronate interconversion	[69]
Body mass reduction comparing pre and post training	US marines on a survival training exercise	Weight	Anxiety severity assessed with GAD-7	Significant correlation between mass reduction and increase in anxiety severity	[73]

OR: odds ratio; CI: confidence interval; HR: Hazard ratio; Q: quartile; *anxiety not defined; GAD: generalised anxiety disorder; ACS: acute coronary syndrome; BMI: Body mass index; CRP: C-reactive protein; DBP: Diastolic blood pressure; eGFR: Estimated glomerular filtration rate; GAD: General Anxiety Disorder; HADS: Hospital anxiety and depression scale; HARS: Hamilton anxiety rating scale; HbA1c: Glycated haemoglobin; HDL: High density lipoprotein; ICD: International classification of diseases; NGAL: Neutrophil gelatinase-associated lipocalin; SBP: Systolic blood pressure; SF-36: Short form-36; WHR: Waist to hip ratio.

### Traditional cardiovascular risk factors

Anxiety has also been associated with poor control of traditional cardiovascular risk factors [75]. Pietrzykowski and colleagues assessed blood pressure, low density lipoprotein cholesterol, body mass index, waist circumference, physical activity, smoking, triglyceride and blood glucose in 200 patients without a history of cardiovascular disease. Anxiety was assessed by HADS. Patients with HADS anxiety scores ≥8 had significantly fewer risk factors controlled than those with HADS anxiety scores <8. Anxiety was associated with larger waist circumference, less physical activity, greater body mass index and serum triglyceride [75]. Stress commonly accompanies anxiety. An analysis of 3267 participants from the St. Jude Lifetime Cohort Study found that stress was associated with increased risk of developing a dysrhythmia (Relative risk, RR, 2.87; 95% CI, 1.21, 6.78) and hypertension (RR 1.42; 95% CI, 1.04, 1.95), while posttraumatic stress syndrome and anxiety were associated with dyslipidemia (RR 1.72; 95% CI, 1.13, 2.62; RR1.54; 95% CI, 1.01, 2.35, respectively) [76]. These findings fit with those Mendelian randomisation studies outlined above pointing to anxiety predisposing to cardiovascular disease via increased traditional risk factors.

## Effect of interventions on anxiety among people with peripheral artery disease

Conventional therapies for anxiety disorder include anxiolytics (e.g. flupirtine), 5-HT reuptake inhibitors (SSRIs e.g. sertraline) and benzodiapines. The effect of these drug therapies and psychological interventions for anxiety has not been studied specifically in patients with PAD, although their benefit in other populations such as people with coronary heart disease and older adults has been established [77,78]. Several studies have examined ways to limit anxiety around the time of peripheral vascular interventions to treat PAD. Several studies have suggested anxiety is elevated prior to peripheral revascularisation and falls following the procedure [6,11]. Banas and colleagues assessed anxiety in 140 patients with PAD using the HADS [11]. Three groups of patients were included: (i) 50 patients with CLTI scheduled for revascularisation; (ii) 50 patients with intermittent claudication schedules for revascularisation; and (iii) 40 patients with intermittent claudication being treated by medical therapy alone. At entry, HADS anxiety scores were significantly greater in patients who were scheduled for revascularisation (Mean [SD] 4.2 [3.6] and 4.2 [3.5] for CLTI and intermittent claudication, respectively) than those being treated by medical therapy alone (1.9 [2.4], *P* < 0.01) [11]. One year after revascularisation the HADS anxiety scores reduced in patients who had revascularisation (2.9 [3.5] and 1.8 [2.2]) and were then like those of patients with intermittent claudication treated medically (1.7 [2.9], *P* > 0.10). The investigators accredited the reductions in HADS anxiety scores to benefits of revascularisation. This interpretation was supported by significant improvement in physical parameters, such as six-minute walking distance and activities of daily living index after revascularisation. It is also possible that patients were more anxious prior to revascularisation because they were concerned about what their outcome of the procedure would be and the reduction at 12 months was a return to baseline levels like those patients not having revascularisation. Fioranelli et al. assessed 138 patients with CLTI undergoing revascularisation in Brazil using the Beck Anxiety and Depression Inventories [6]. Immediately prior to revascularisation, 29.7%, 17.4%, and 8.0% of patients had mild, moderate and severe anxiety, respectively. Thirty days after revascularisation, the proportion of patients with mild, moderate and severe anxiety reduced to 17.2%, 7.2%, and 0.9%, respectively. However, by 6 months the proportion of patients with mild, moderate and severe anxiety increased to baseline levels of 32.4%, 5.4%, and 8.1% [6].

Table 6 illustrates the findings from several randomised controlled trials testing interventions to reduce anxiety in patients with PAD [25]. In a 204-person randomised trial listening to classical music via headphones during an endovascular revascularisation procedure to treat CLTI significantly reduced anxiety assessed by numerical rating scale, as compared to controls [22]. Multiple randomised trials of different exercise regimens or health coaching to increase home exercise have reported significant reductions in anxiety as compared to controls (Table 6) [20,25,32]. Similarly, a cluster randomised controlled trial found that a comprehensive secondary prevention program involving education and assistance to control modifiable risk factors through medication and lifestyle changes led to significantly less anxiety in patients exposed to the intervention [12]. In contrast, a small, randomised trial of a behaviour change intervention in addition to a home-based exercise program failed to reduce anxiety significantly as compared with the home exercise program alone [27]. Similarly, a trial of reviewing peripheral artery imaging during consent for revascularisation and a trial testing the drug naftidrofuryl found no significant benefit of the interventions on anxiety severity [18,29]. These findings support the use of exercise rehabilitation to treat PAD and reduce anxiety. They also highlight the need for further research to identify the most effective ways to reduce anxiety around the time of peripheral revascularisation to treat PAD.

**Table 6:** Effect of different interventions on anxiety in patients with peripheral artery disease in randomised controlled trials

Population	Number	Intervention	Control	Study design	Effect on symptoms of		Reference
					anxiety	depression	
Intermittent claudication	1982	Telephone health (exercise) coaching (9 sessions) with remote physical activity monitoring	Usual care	Open-label RCT	0.41 (0.07, 0.76)*	0.67 (1.07, 0.28)*	[25]
Intermittent claudication or CLTI having percutaneous transluminal angioplasty	204‡	Listening to classical music via headphones during endovascular treatment	No music	Open-label RCT	Anxiety NRS during the procedure significant lower in intervention compared to control group	NR	[22]
Intermittent claudication	56	Twelve week arm supervised ergometry exercise therapy	Twelve week supervised treadmill exercise therapy	Open-label RCT	0.21 (−1.03, 1.44; *P* = 0.741)**	0 (−1, 2; *P* = 0.509)**	[20]
Intermittent claudication	29	Eight week home calf raising exercise program	Eight week home walking exercise program	Open-label RCT	Significantly reduced anxiety in the intervention group#	NR	[32]
Diagnosed with ischemic heart disease, stroke or PAD	1224†	Comprehensive secondary prevention program in primary care: Education and assistance to control modifiable risk factors through lifestyle changes and drug therapies	Usual care	Cluster RCT	Intervention group less anxiety at final follow-up than control group (*P* = 0.05)††	Intervention group less depression symptoms at final follow-up than control group (*P* = 0.02)††	[12]
Intermittent claudication	754‡‡	Naftidrofuryl	Placebo	Exploratory analysis of 3 placebo-controlled RCTs	No effect on disease specific anxiety assessed by CLAU-S	Significant improvement in mood assessed by CLAU-S	[29]
Patients undergoing revascularisation for PAD or AAA repair	51	Consent discussion with review of imaging	Consent discussion with no review of imaging	Open-label RCT	No significant effect of intervention	No significant effect of intervention	[18]
Intermittent claudication	73	Home exercise program with behaviour change intervention supported by a smartphone application	Home exercise program without behaviour change support	Open-label RCT	No significant between group differences	No significant between group differences	[27]

*Mean (95% confidence intervals) difference in Generalised anxiety disorder-7 scale or Patient health questionnaire-9 scores at 12 months (differences were not significant at 24 months); ‡49 patients excluded post randomisation; ‡‡ 45 patients excluded post randomisation; CLTI: chronic limb threatening ischemia; NRS: numerical rating scale; ** HADS: hospital anxiety and depression scale; # no information provided on how assessed and comparison between groups; NR: not reported; PAD: peripheral artery disease; † only 79 (6.5%) had PAD; ††assessed with Goldberg anxiety-depression scale; CLAU-S: disease-specific quality of life tool for intermittent claudication; AAA: abdominal aortic aneurysm.

**Figure 4:**
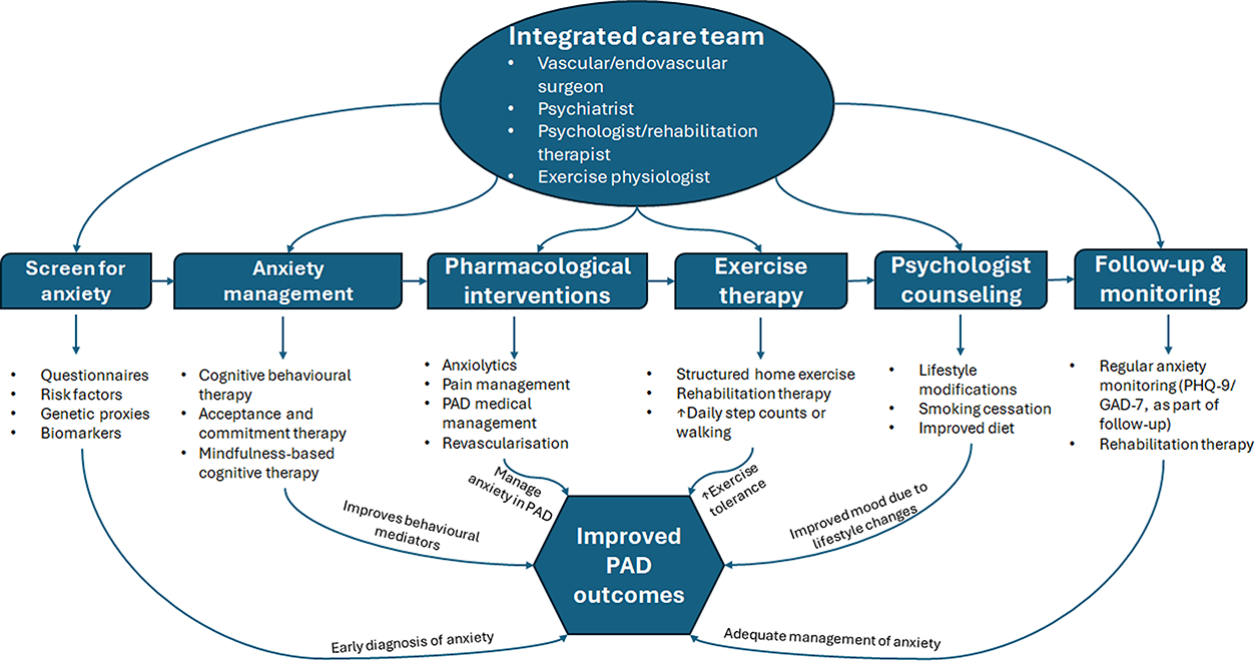
Integrated care program for anxiety management in PAD patients. Questionnaires (HADS-A, GAD-2, GAD-7, PHQ-9). Integrated care program for anxiety management in PAD patients. Questionnaires (HADS-A, GAD-2, GAD-7, PHQ-9). Risk factors: smoking, coronary heart disease, hypertension, smoking, minimum family income. Biomarkers: C-reactive protein, neutrophil-lymphocyte ratio, tissue inhibitor of metalloproteinase-4, Neutrophil gelatinase-associated lipocalin, haemoglobin, brain-derived neurotropic factor. PAD medical management: blood pressure control, glucose control, lipid control, anti-platelet prescriptions.

We propose an integrated care program that emphasises early diagnosis, exercise therapy, behavioural therapy and psychological support, risk factor modifications, and mechanisms involved to address the understudied role of anxiety in people with PAD (Figure 4). Cognitive Behaviour Therapy (CBT) is a first-line treatment for anxiety, with emerging third-wave therapies such as acceptance and commitment therapy (ACT) and mindfulness-based cognitive therapy (MBCT) showing therapeutic potential for psychological flexibility, emotion regulation, and improved self-care behaviours in patients with cardiovascular disease [79,80]. However, no randomised trials have evaluated their efficacy in PAD patients. Given the existing evidence from related cardiovascular diseases, integrated behavioural care holds potential to improve outcomes in PAD patients with anxiety. We recommend prioritising PAD-specific trials to: (i) establish whether anxiety directly influences PAD progression, (ii) compare the efficacy of CBT, ACT, MBCT, and anxiolytics against conventional medical management in PAD patients with anxiety, and (iii) evaluate the impact of integrated care on clinical outcomes and quality of life. Until such evidence is available, treatment recommendations should remain cautious. Nevertheless, insights from other cardiovascular disease could be leveraged while acknowledging the unique needs of PAD patients.

## Limitations

This review has several limitations. While every attempt was made to include relevant articles, this review did not involve a systematic search and thus it is possible that important studies were not included. Critical analysis of the included studies was performed but no formal risk of bias assessment was undertaken. Our search focused on the last 5 years to be contemporary, meaning important studies published prior to this may have been missed. Importantly, the included studies largely focused on symptoms of anxiety assessed with questionnaires. None of the studies reported different types of anxiety disorders and this requires assessment in future studies.

## Conclusions

This review highlights that anxiety is very common in people with PAD. Anxiety appears to be heighted around the time of revascularisation procedures and associated with increased risk of complications. Suggested mechanisms by which anxiety increases the risk of complications include activations of stress associated neurophysiological responses, sub-optimal control of traditional risk factors and effects on biomarkers implicated in cardiovascular disease. There is some evidence to support that exercise therapy and other secondary prevention programs reduce anxiety. Further research is needed to understand the consequences of anxiety in people with PAD and identify effective treatments.

## Clinical perspectives

- Prevalence of anxiety disorder among PAD patients undergoing revascularisation had approximately doubled between 2011 and 2017.
- Female sex, financial concerns, and lack of social support were key risk factors for anxiety in PAD patients.
- PAD-specific trials are needed to establish whether anxiety directly influences PAD progression.
- Integrated care models may be beneficial for anxious patients with PAD. Until such evidence is available, treatment recommendations for PAD should remain cautious.

## Data Availability

All presented data is available from publicly available databases.

## Open Access

Open access for this article was enabled by the participation of James Cook University in an all-inclusive Read & Publish agreement with Portland Press and the Biochemical Society under an agreement with CAUL.

## Abbreviations

ACT: Acceptance and commitment therapy
CLTI: Chronic limb threatening ischemia
GAD: Generalised anxiety disorder
HADS: Hospital anxiety and depression scale
HARS: Hamilton anxiety rating scale
HPA: Hypothalamic-pituitary-adrenal
ICD: International Classification of Diseases
ICHOM: International Consortium for Health Outcomes Measurement
MBCT: Mindfulness-based cognitive therapy
REML: Restricted maximum likelihood
PAD: Peripheral artery disease
PAQ: Peripheral artery questionnaire
